# Reviewing the Role of the Efferent Vestibular System in Motor and Vestibular Circuits

**DOI:** 10.3389/fphys.2017.00552

**Published:** 2017-08-02

**Authors:** Miranda A. Mathews, Aaron J. Camp, Andrew J. Murray

**Affiliations:** ^1^Sensory Systems and Integration Laboratory, Bosch Institute, Discipline of Biomedical Science, University of Sydney Sydney, NSW, Australia; ^2^Sainsbury Wellcome Centre for Neural Circuits and Behaviour, University College London London, United Kingdom

**Keywords:** efferent vestibular system, efferent vestibular nucleus, EVS, EVN, corollary discharge, VOR, vestibular, vestibular plasticity

## Abstract

Efferent circuits within the nervous system carry nerve impulses from the central nervous system to sensory end organs. Vestibular efferents originate in the brainstem and terminate on hair cells and primary afferent fibers in the semicircular canals and otolith organs within the inner ear. The function of this efferent vestibular system (EVS) in vestibular and motor coordination though, has proven difficult to determine, and remains under debate. We consider current literature that implicate corollary discharge from the spinal cord through the efferent vestibular nucleus (EVN), and hint at a potential role in overall vestibular plasticity and compensation. Hypotheses range from differentiating between passive and active movements at the level of vestibular afferents, to EVS activation under specific behavioral and environmental contexts such as arousal, predation, and locomotion. In this review, we summarize current knowledge of EVS circuitry, its effects on vestibular hair cell and primary afferent activity, and discuss its potential functional roles.

## Introduction

The flow of information through the nervous system can be viewed as relatively simple. Sensory stimuli from the external environment activates peripheral receptors and, in turn, sensory afferent neurons. Alteration in firing patterns of sensory afferents encodes information used by the central nervous system to modulate motor output. This linear description of nervous system function is complicated by the central recruitment of efferent pathways, which innervate peripheral sensors and modulate their activity. When considered in the context of sensory information, efferent pathways provide the nervous system with the ability to adjust its own view of the external environment. This type of efferent modulation is common in many vertebrate sensory systems, some examples of which include olfactory efferents in the pigeon (Atoji and Wild, 2014), somatosensory efferents in the rat (Zakiewicz et al., 2014), and retinal efferents in primates (Ortiz et al., 2017).

A classic example of efferent modulation of sensory inflow can be found in the auditory system. Auditory efferents (the olivocochlear system) have been implicated in noise protection, sound localization, and the ability to discriminate signal from background noise (Kawase and Liberman, 1993; Kawase et al., 1993; Guinan, 2006, 2010; Ciuman, 2010). This latter function of signal extraction could be used as a means of filtering self-generated sounds (Tomchik and Lu, 2006). For example, the medial olivocochlear bundle (MOC) has been shown to restore the dynamic range (cochlear gain) and ensuing signal responsiveness of the auditory nerve (Giraud et al., 1997; Liberman and Guinan, 1998), probably in a context dependent manner (for example during attention and experience; de Boer et al., 2012), or in accordance with signals generated from cortical (auditory) regions (Xiao and Suga, 2002). This “anti-masking” hypothesis effectively enhances the perception of noise, for example from self-generated sound, in a noisy environment (for review, see Guinan, 2006). Other efferent systems exist in the somatosensory system, where feedback from muscle spindles can be modulated by Gamma (fusimotor) motor neurons. This efferent projection may sensitize primary afferents to detect changes in muscle length, as well as deviations from intended movement, and extend the dynamic range of spindle responses (Burke et al., 1979; Ellaway et al., 2015).

In general, efferent neurons are reasonably well characterized in terms of their anatomy, physiology, and molecular properties, and most efferent systems have at least a putative function ascribed to them. The efferent vestibular system (EVS) however has consistently received less attention, and as such, its direct function has been difficult to define. The potential functional significance of the EVS is underscored by the fact that all vertebrates possess some form of a vestibular efferent system (Meredith, 1988). However, despite previous physiological and anatomical studies a conclusive functional role in mammals remains unclear. Here, we briefly summarize current knowledge concerning the anatomy, pharmacology, and physiological actions of the EVS. The main purpose of this publication is however to discuss potential mammalian functional roles within sensorimotor circuits, brought about by a review of relevant literature, with a particular focus on recent work. Although this review focuses on labyrinth efferents, lateral line efferents are also indirectly addressed when considering non-mammalian species, particularly given evidence for common lateral line and labyrinth efferents in the frog (see Hellmann and Fritzsch, 1996).

## Anatomy and morphology of the EVS across vertebrates

The vestibular labyrinth provides the nervous system with information regarding head and body movement in space. This sensory information plays a critical role in our ability to interact with the environment through our capacity for coordinated motor actions, maintenance of balance, and spatial navigation. Briefly, the vestibular component of this process includes the generation of the initial movement signal by the activation of hair cells in the semicircular canals and/or otolith organs in the inner ear, and reaches the brain via primary sensory afferents. Efferent projections arise from the brainstem and project to the periphery, forming synapses with vestibular hair cells and afferents. For a detailed review of this neuroanatomical organization including cell body and dendritic morphology, axonal pathways to the periphery, and peripheral branching patterns, see Holt et al. (2011).

As mentioned above, all vertebrates possess some form of a vestibular efferent system (Meredith, 1988; Highstein, 1991), central neurons of which derive from a similar developmental origin (for example rhombomere 4 in mice, and 4 and 5 in chicken; see Hellmann and Fritzsch, 1996). In mice, efferent vestibular nucleus (EVN) neurons express high levels of the transcription factors *Gata2* and *Gata3* (Tiveron et al., 2003). Knockout of *Hoxb1* (which controls the expression of *Gata2* and *Gata3*) prevents the development of EVN neurons, along with populations of spinally projecting cells in the medial and lateral vestibular nuclei (Di Bonito et al., 2015).

**Figure 1:**
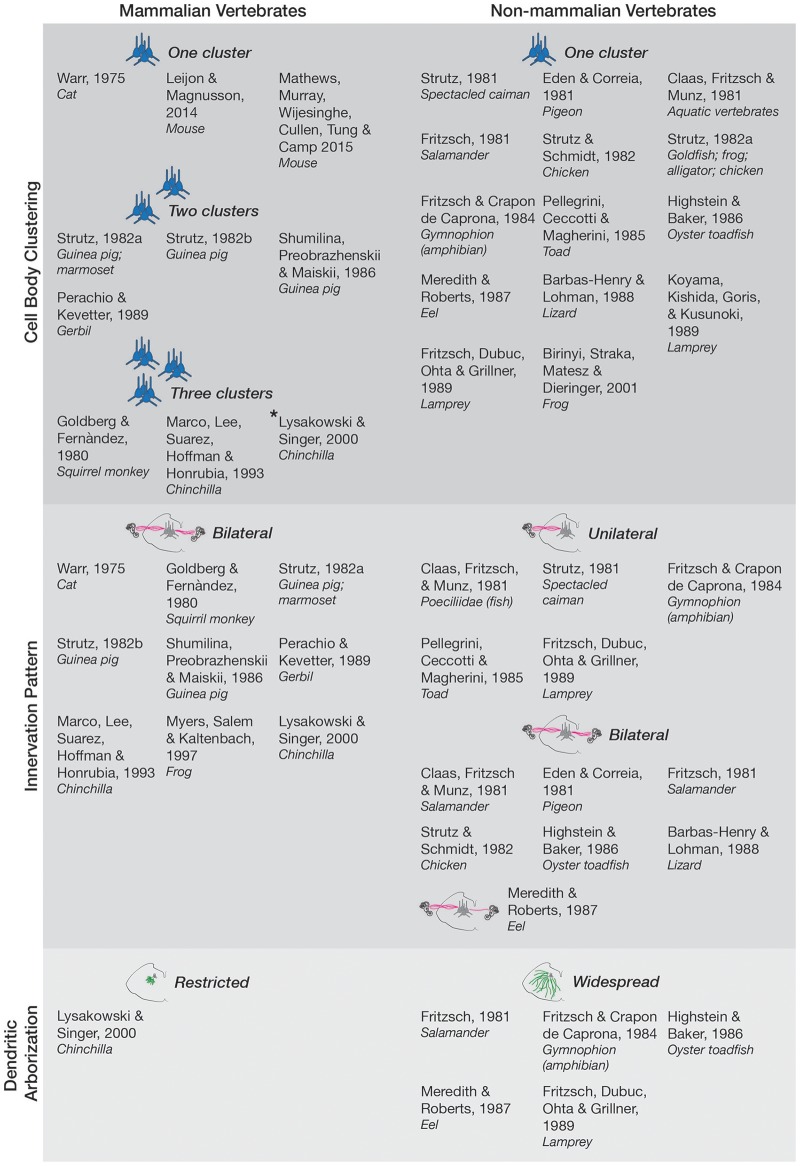
Anatomy and morphology of the EVS across vertebrates. Studies that directly investigated EVS anatomy and morphology were separated under the following categories-cell body clustering, innervation pattern, and dendritic arborization. Studies that assessed more than one category are mentioned in each respective category they investigated. Where more than one cell body cluster was observed, the number of clusters is labeled and depicted with the respective number of blue pictorial clusters. Asterisk next to Lysakowski and Singer (2000) denotes one cluster likely projecting to middle ear instead of peripheral vestibular labyrinth. Uni- and bilateral projections are also labeled and depicted with pink lines from a coronal brainstem schematic out towards the inner ear (drawings not to scale). Only one bilateral projection is drawn for Meredith and Roberts (1987) eel as they denoted it as a minor finding. Expansive green lines along the brainstem tegmentum denote widespread arborization of dendrites, and shorter green lines depict restricted arborization, as labeled. Nonmammalian species included all animals groups not classified as mammals.

The position of efferent vestibular neurons within their final brainstem nuclei is remarkably conserved across species. This conservation of both system and anatomical localization hints toward a common function of this circuit. However, there appears to be three main phylogenetic modifications of the EVN as we transition from non-mammalian to mammalian vertebrates (summarized in Figure 1).

### Cell body clustering

In non-mammalian vertebrates efferent neurons are confined to a single cluster (Claas et al., 1981; Eden and Correia, 1981; Fritzsch, 1981; Strutz, 1981, 1982a; Strutz and Schmidt, 1982; Fritzsch and Crapon de Caprona, 1984; Pellegrini et al., 1985; Highstein and Baker, 1986; Meredith and Roberts, 1987; Barbas-Henry and Lohman, 1988; Fritzsch et al., 1989; Koyama et al., 1989; Birinyi et al., 2001). However, the mammalian EVS can possess multiple groups, dependent on species. Mouse and cat have only one cluster of EVN neurons, located near the facial nerve genu (Warr, 1975; Leijon and Magnusson, 2014; Mathews et al., 2015). In other mammalian studies, more than one cluster was observed with the major nucleus being referred to as group e (Goldberg and Fernàndez, 1980), located dorsal and/or ventral to the facial nerve (Shumilina et al., 1986; Perachio and Kevetter, 1989). Smaller clusters are scattered in the caudal pontine reticular nucleus and the medial reticular nucleus (Strutz, 1982a,b). Interestingly, in the chinchilla, three anatomically distinct groups near the facial nerve, abducens nerve, and vestibular nuclei were distinguished (Marco et al., 1993; Lysakowski and Singer, 2000), though the cluster ventral to the facial nerve likely reflects projections to the middle ear rather than the peripheral vestibular labyrinth (Lysakowski and Singer, 2000).

### Peripheral innervation pattern

The mammalian EVN projects bilaterally in the chinchilla (Marco et al., 1993; Lysakowski and Singer, 2000), guinea pig (Strutz, 1982a,b; Shumilina et al., 1986), squirrel monkey (Goldberg and Fernàndez, 1980), marmoset (Strutz, 1982a), gerbil (Perachio and Kevetter, 1989), and cat (Warr, 1975). Non-mammalian vertebrates on the other hand have a heterogeneous projection profile. For example, the oyster toadfish (Highstein and Baker, 1986), pigeon (Eden and Correia, 1981), chicken (Strutz and Schmidt, 1982), salamander (Claas et al., 1981; Fritzsch, 1981), lizard (Barbas-Henry and Lohman, 1988), eel (Meredith and Roberts, 1987), and frog (Myers et al., 1997), project bilaterally, while the spectacled caiman (Strutz, 1981), toad (Pellegrini et al., 1985), poeciliidae (fish) (Claas et al., 1981), lamprey (Fritzsch et al., 1989), and the amphibian gymnophion (Fritzsch and Crapon de Caprona, 1984) project unilaterally. The functional reasons why mammalian (and some non-mammalian vertebrate) neurons require the ability to modulate vestibular input bilaterally though is unclear.

### Dendritic arborization

Dendritic arbors of efferent neurons are spread out across the brainstem tegmentum, and also cross to the contralateral side in animals such as the lamprey, toadfish, eel, salamander, and amphibian (Fritzsch, 1981; Fritzsch and Crapon de Caprona, 1984; Highstein and Baker, 1986; Meredith and Roberts, 1987; Fritzsch et al., 1989). In the lamprey and other non-mammalian vertebrates, this widespread arborization could serve as a means of obtaining information from both the ipsi- and contralateral CNS that can then be used to modulate peripheral action bilaterally despite unilateral efferent projection. This mechanism could reflect an early approach to bilateral efferent modulation given the phylogenetic age of the lamprey that precedes mammals (Neidert et al., 2001). In chinchilla however, their dendritic arbors are significantly more compact (Lysakowski and Singer, 2000). The relatively sparse studies of dendritic arborization in different species though make it difficult to extrapolate whether this reduction in arborization is ubiquitous. This information is useful though, because dendritic patterns and arbor could reflect specific transcription factors, cell surface receptors, various cytoskeletal elements and pathways, as well as interactions between synaptically connected neurons (reviewed in Jan and Jan, 2010).

## Vestibular afferent and hair cell responses to EVS activation

**Figure 2:**
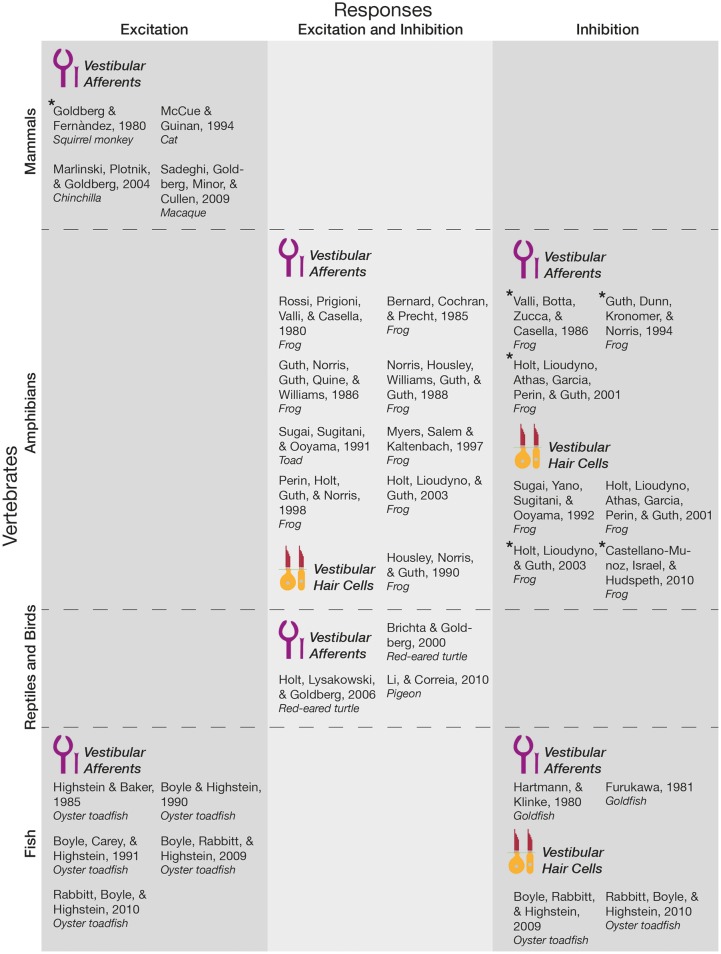
Responses of primary vestibular afferents and hair cells to EVS activation and ACh application. Studies that electrically activated the EVS (i.e., efferent fibers at the level of the vestibular nerve or the EVN itself), or applied acetylcholine (ACh) at the vestibular labyrinth, before measuring the responses of vestibular hair cells or primary afferents were included in this Figure. They were categorized along the vertebrate scale, whether they measured vestibular hair cell or afferent responses, and the nature of the response itself. Asterisk denotes papers where excitation or inhibition was stated by the authors as a minor response subset. As labeled in this Figure, calyx and boutons endings of primary vestibular afferents are drawn in purple, while type I and II hair cells are drawn in yellow and red. These simplified illustrations do not represent the anatomical differences between species; instead reflect general peripheral targets (i.e., hair cells or primary afferents).

Below, the literature concerning how primary vestibular afferents and hair cells respond to vestibular efferent activation is summarized (see also Figure 2).

A majority of non-mammalian, and all mammalian vertebrates (i.e., fish and mammals, respectively) demonstrate increases in background afferent discharge in response to stimulation of the efferent pathway. These effects are particularly pronounced in irregularly firing afferents and may result in a concomitant reduction in sensitivity or gain (Goldberg and Fernàndez, 1980; Highstein and Baker, 1985; Boyle and Highstein, 1990; Boyle et al., 1991, 2009; McCue and Guinan, 1994; Marlinski et al., 2004; Sadeghi et al., 2009; Rabbitt et al., 2010), similar to gain control mechanisms observed in other efferent systems including the MOC described above (see Introduction). While the predominant effect is afferent excitation in these animals, Goldberg and Fernàndez (1980) also observed inhibitory responses in three afferent fibers in the squirrel monkey. Although this recording represented <1% of their total recordings, it may hint toward heterogeneity of EVN cell types, particularly since heterogeneity of afferent response to EVS activation is found in other species.

Other such species where heterogeneous afferent responses were found include red-eared turtles and amphibians, where both excitatory and inhibitory afferent responses were routinely observed; suggesting that the reduced number of inhibitory responses found in mammals may represent an evolutionary modification. It is notable that these responses were dependent on the location of the afferent fiber within the crista (Rossi et al., 1980; Bernard et al., 1985; Valli et al., 1986; Sugai et al., 1991; Brichta and Goldberg, 2000; Holt et al., 2006). In the frog, ipsilateral efferent-mediated effects were predominantly excitatory (92% of recordings), while contralateral efferent-mediated effects were inhibitory (95% of recordings; Myers et al., 1997). Moreover, this efferent-mediated afferent inhibition is dependent on the release of acetylcholine (ACh) at efferent post-synaptic terminals (Rossi et al., 1980), the application of which at the vestibular labyrinth mimics stimulation of the efferent fibers and produces both inhibitory and excitatory effects on afferent fibers (Guth et al., 1986; Norris et al., 1988; Holt et al., 2003), though inhibition was predominant in saccular afferents while excitation was predominant in semicircular canal afferents (Guth et al., 1994; Perin et al., 1998; Holt et al., 2001, 2003). Interestingly, efferent activation induces larger excitation responses in irregular, than regularly firing afferents (Marlinski et al., 2004), that can be decomposed into fast (10–100 ms kinetics), and slow (declines over several seconds following an initial build) components (Goldberg and Fernàndez, 1980; Brichta and Goldberg, 2000). Further, in goldfish excitatory afferent post-synaptic potentials (EPSPs) were shown to be abolished (Furukawa, 1981), and semicircular canal afferent discharges were tonically inhibited (Hartmann and Klinke, 1980) following efferent stimulation, suggesting that at least in some species inhibition predominates central-peripheral synaptic transmission.

Despite a relative scarcity of direct hair cell recordings, there is a general consensus that vestibular efferent stimulation elicits inhibition in target hair cells (Sugai et al., 1992; Boyle et al., 2009; Castellano-Munoz et al., 2010; Rabbitt et al., 2010). This is surprising particularly because excitation has been observed in a minority of hair cells (Castellano-Munoz et al., 2010), and efferent projections are extremely divergent to both hair cells and afferents (Goldberg et al., 1990; Lysakowski and Goldberg, 1997). Moreover, application of cholinergic agonists and antagonists that activated mAChRs or nAChRs on vestibular hair cells produced excitatory and inhibitory effects on the membrane potential in the pigeon (Li and Correia, 2011). Cholinergic excitation was also observed in the frog (Housley et al., 1990; Holt et al., 2003). Nonetheless, this consensus presumably stems from the neurotransmitter and receptor composition used by the EVS (for a detailed review of EVN neurotransmitter profile, see Holt et al., 2011). In particular, there is ample evidence for the presence of acetylcholine in EVN neurons (Schwarz et al., 1986; Perachio and Kevetter, 1989; Ishiyama et al., 1994), as well as in the terminals in the sensory epithelium of vestibular end organs (Hilding and Wersall, 1962; Kong et al., 1994; Matsuda, 1996). The release of ACh, and activation of nicotinic ACh receptors at the efferent synapse produces inhibitory post-synaptic potentials in hair cells of the inner ear and cochlea (Art et al., 1984; Sugai et al., 1992; Elgoyhen et al., 1994, 2001; Goutman et al., 2005). As previously described though, ACh has also been implicated in excitatory (Rossi et al., 1980; Bernard et al., 1985; Sugai et al., 1991; Holt et al., 2015), as well as inhibitory (Holt et al., 2001, 2006) responses evoked in vestibular afferents by efferent stimulation.

There is evidence that this heterogeneity of afferent and hair cell responses by efferent stimulation across species is related to distinct post-synaptic cholinergic receptors at efferent synapses (for a detailed review, see Jordan et al., 2013). For example, efferent-mediated activation of α9/10 nicotinic ACh receptors (nAChR) in type II hair cells underlies their inhibition (Elgoyhen et al., 1994, 2001; Oliver et al., 2000; Weisstaub et al., 2002), while α6β2 nAChRs have been implicated in afferent excitation (Holt et al., 2015). Moreover, the kinetics of slow afferent excitation suggests that muscarinic AChRs (mAChRs) mediate this response (reviewed in Jordan et al., 2013). Indeed recent work in turtles demonstrated mAChR activation during efferent-mediated slow excitatory responses of primary vestibular afferents (Holt et al., 2017).

Establishing a neurotransmitter and receptor profile for the EVN is helpful in formulating an improved understanding of its function. EVN neurons are generally accepted to be cholinergic but also express calcitonin gene-related peptide (CGRP; Perachio and Kevetter, 1989; Tanaka et al., 1989; Ohno et al., 1991; Wackym et al., 1991). Substances commonly co-expressed in cholinergic neurons, or those that parallel auditory efferents may also be present in EVN neurons. For example, adenosine 5′-triphosphate (ATP) which is commonly released together with ACh, noradrenaline, dopamine (DA) and GABA (Abbracchio et al., 2009), has also been shown to depolarize vestibular hair cells (Rennie and Ashmore, 1993; Aubert et al., 1994, 1995; Rossi et al., 1994). Likewise, neuronal nitric oxide synthase (nNOS) that synthesizes nitric oxide has been localized in EVN neurons as well as their terminals within the end organs (Lysakowski and Singer, 2000; Takumida and Anniko, 2002; Desai et al., 2005). Opioid receptors have also been implicated in excitatory post-synaptic input to vestibular afferents (Andrianov and Ryzhova, 1999; Vega and Soto, 2003) and inhibitory pre-synaptic input to hair cells (Vega and Soto, 2003). Together, these studies highlight the complexity of central-peripheral vestibular interactions and again hint at diversity in functional output of the EVS both within and across species.

## Physiology of EVN neurons

To date, few studies have directly recorded from neurons within the EVN either *in vitro* or *in vivo*. Marlinsky (1995) recorded the extracellular discharge activity of EVN neurons in response to semicircular canal stimulation as well as from their neighboring medial vestibular nucleus (MVN) neurons in decerebrate and decerebellate guinea-pigs. Paralleling MVN neuron responses, a majority of EVN neurons were antidromically excited by ipsilateral but not contralateral canal stimulation, suggesting direct vestibular afferent input (Marlinsky, 1995). A minor subset of EVN neurons also responded with an increased activity to contralateral canal stimulation and contralateral tilt, which was also observed in recorded MVN neurons (Marlinsky, 1995). Importantly, neurons were classified as vestibular efferents if they were antidromically excited following electrical stimulation of the anterior semi-circular canal, while cells that were monosynaptically activated were classified as MVN neurons. Although, this study implicates the EVN in mediating changes to contralateral vestibular nuclei interactions that occur at the level of peripheral sensors, the classification of recorded neurons was not conclusive (for example, retrograde staining of recorded neurons was not performed to confirm their locations), and therefore provides a caveat to their results. However, in addition to canal stimulation, a significant increase in the discharge rate of 40% of peripheral efferent fibers was observed in response to sciatic nerve stimulation in the frog (Caston and Bricout-Berthout, 1984), suggesting that the function of the EVN may be linked to the spinal circuits underlying locomotion, a potential functional role elaborated further in Section The EVS Signals Corollary Discharge between Vestibular and Other CNS Structures.

More recent work has investigated the physiological characteristics of EVN neurons including passive membrane properties, discharge profiles, and synaptic input profile. Cholinergic EVN neurons were identified in one study using transgenic mice engineered to express enhanced green fluorescent protein (eGFP) under the choline-acetyltransferase (ChAT) promotor (Leijon and Magnusson, 2014). In another study, the location of EVN neuronal recordings were confirmed using retrograde tracing from the posterior semicircular canal in *ChAT:: tdTomato* transgenic mice which confirmed the location of cholinergic EVN neurons, and the staining of each recorded neuron (Mathews et al., 2015). Both studies were conducted *in vitro*. EVN neurons displayed a negative membrane potential and characteristic firing pattern (Leijon and Magnusson, 2014; Mathews et al., 2015), that appear to be mediated by fast transient outward K^+^ currents (*I*_A_) (Leijon and Magnusson, 2014). EVN neurons demonstrated two discharge profiles at rest—spontaneous and non-spontaneously active (Highstein and Baker, 1985; Mathews et al., 2015), perhaps indicating a neuronal heterogeneity within the EVN. When compared to lateral olivocochlear (LOC) neurons (as recorded in Leijon and Magnusson, 2014)—typified with a long first spike latency followed by tonic firing—vestibular efferent neurons displayed onset burst spiking followed by sparse firing (Leijon and Magnusson, 2014; Mathews et al., 2015), indicating distinct functional roles of both inner-ear projecting brainstem nuclei. However, the discharge profile of these EVN neurons bears a striking resemblance to earlier electrophysiological recordings of rat LOC neurons in response to depolarising current steps from a hyperpolarized membrane potential (Fujino et al., 1997). If these nuclei share similar firing patterns, it could suggest a common functional role. For example, in mice where the LOC system was lesioned, sound localization in space was impaired indicating a role for the LOC system in balancing interaural cues (Darrow et al., 2006). Given the ipsilateral and contralateral projection patterns of the EVN (see Figure 1) a similar function in balancing vestibular asymmetries may also be important for modulating bilateral vestibular sensitivity.

Neighboring brainstem MVN neurons on the other hand have been characterized by tonic discharge (Beraneck et al., 2003; Camp et al., 2006, 2010), known to code for intensity of inputs. Burst firing similar to that seen in the EVN has previously been demonstrated to serve as a “wake-up” call during dormancy in response to novel stimuli—it has been suggested to be advantageous in improving stimulus detectability and enhancing cortical activation (Weyand et al., 2001; Sherman, 2005; Llinas and Steriade, 2006). It is plausible for EVN neurons to generate short bursts in response to various synaptic inputs that serve to modulate peripheral targets, and thus aid in quick vestibular accommodation.

EVN neurons also demonstrate a high gain, or sensitivity to inputs, when compared with MVN neurons (Mathews et al., 2015)—they appear better suited to responding rapidly to changes in synaptic strength (i.e., inputs). But what drives EVN activation? It appears that individual EVN neurons receive exclusively excitatory or inhibitory inputs, or a combination of both (mixed), although excitatory inputs predominate (Mathews et al., 2015). This suggests that the homogenous output of EVN neurons is governed by an excitatory drive from other parts of the CNS, at least in mice *in vitro*. While the afferent input system demonstrates properties consistent with a feedback loop (Plotnik et al., 2005), other direct connections to the EVN have yet to be identified. A study that exploited pseudorabies virus to trace polysynaptic inputs uncovered inputs from autonomic centers including the hypothalamus, reticular formation, solitary nucleus, raphe nucleus, as well as from other vestibular nuclei and areas in the motor cortex (Metts et al., 2006). Establishing a map of direct monosynaptic partners of the EVN could shed light on the context in which this nucleus is activated as well as its role in the central processing of balance and coordination.

## Functional role/s of the EVS

The first studies of the vestibular efferent system began in the 1960s, but despite over 50 years worth of physiological recordings, anatomy, and pharmacology, its mammalian functional role remains contentious. There are several broad, and in many cases overlapping hypotheses based on the fundamental vestibular efferent action of modulating hair cell and primary sensory afferent firing. These broad hypotheses are described below, and include:

- Differentiation of active and passive movements
- Context dependent modulation of vestibular sensitivity
- Top-down modulation of vestibular sensitivity
- Efference copies of spinal motor commands
- Participation in vestibular plasticity.

### The EVS differentiates between active and passive motion

One of the most popular suggestions surrounding EVS function concerns the neural anticipation of volitional head movements. It is understood that EVN neurons receive inputs and respond to activation from semicircular canals (Schmidt, 1963; Precht et al., 1971; Blanks and Precht, 1976), and otolith organs (Klinke and Schmidt, 1968) from both ears. Efferent activation of afferent fibers is non-uniform across vestibular end-organs (Plotnik et al., 2002), and relative to afferent sensitivity (Highstein, 1992)—such that low-gain afferents are minimally affected while high-gain afferents are profoundly affected. This differential activation of afferents that is specific to the context could potentially contribute to the mechanisms used to differentiate the responses (e.g., occulomotor) to volitional vs. passively-applied movement.

Indeed, an attractive early hypothesis implicated the EVS in anticipation of volitional head movement and ensuing gaze shift, by improving the dynamic range of primary vestibular afferents (Goldberg and Fernàndez, 1980; Highstein, 1991; Brichta and Goldberg, 2000). However, no changes were found in the resting discharge of afferent fibers between passive, and active head and eye movements in alert macaques under normal conditions (Cullen and Minor, 2002) or after labyrinthectomy (Sadeghi et al., 2007). For example, extracellular, single-unit semicircular canal afferent recordings were comparable during passive head rotations, as well as during volitional (including gaze shift and pursuit) movements (Cullen and Minor, 2002). This has also been demonstrated for passive and active linear head movements (Jamali et al., 2009). Moreover, following unilateral labyrinthectomy (UL), no differences were observed in canal afferent sensitivity or phase—suggesting that they do not play a role is vestibular compensation following labyrinthectomy (Sadeghi et al., 2007). This role of the EVS in vestibular plasticity and compensation is discussed in Section The Role of the EVS in Vestibular Plasticity. Notably though, UL in macaques resulted in a decrease in the proportion of regular and increase in the proportion of irregular afferent fibers (Sadeghi et al., 2007; Yu et al., 2014), while in contrast α9-knockout mice displayed an increase in the proportion of regular and decrease in the proportion of irregular afferent fibers (Han et al., 2007). Therefore, it is plausible that the lack of change in sensitivity during compensation observed by Sadeghi et al. (2007) could in fact be masked by changes in the distribution of afferent discharge properties following UL. Overall, it appears that the EVS serves a more complex role in motor and postural coordination than simply coding active vs. passive head movements, which represents only one example of context dependent modulation of peripheral vestibular sensitivity.

### The EVS modulates vestibular sensitivity in a context dependent manner

Previous work has shown that vestibular reflexes can be altered depending on environmental or behavioral context, such as during a perceived threat to balance (Lim et al., 2016; Naranjo et al., 2016). Pressure applied to skin, passive limb movement, visual stimulation, as well as states of arousal and predation have all been demonstrated to activate the EVS (Schmidt, 1963; Klinke, 1970; Precht et al., 1971; Highstein and Baker, 1985; Highstein, 1992). For example, Highstein (1992) showed that efferent neurons display a low (4–5 spikes/second), irregular discharge frequency when the animal is at rest. Multimodal stimulation including light touch, sound, visual stimuli as well as vestibular sensation effectively increased efferent discharge (Highstein and Baker, 1985; Highstein, 1992), however response decay times varied between 100 and 600 ms (Highstein and Baker, 1985). This efferent “activation” was accompanied by a proportionally increased level of animal arousal (Highstein, 1992). Locomotor activity could underlie the described efferent activation under different stimuli and interestingly, a recent study using tadpoles (Chagnaud et al., 2015) implicated the EVS in corollary discharge from locomotion. However, given that the oyster toadfish preparations were spinalized, this cannot be inferred directly from this work. Highstein (1992) also supports the hypothesis that efferent activation could serve as a “wake up call” (see Section Physiology of EVN Neurons)—increasing firing in neurons sequentially associated with vestibular processing. In addition, electrical activation of vestibular efferents results in behavior typical of aroused free-swimming toadfish (Highstein, 1992). These studies highlight the idea that the EVS is activated under a diverse suite of contexts and an understanding of these contexts will be informative for understanding specific functional roles of the EVS.

If vestibular efferents are activated following both vestibular and non-vestibular stimulation, it is interesting to speculate whether the EVN could influence or be influenced by other systems implicated in vestibular labyrinth activity, such as during vestibular control of sympathetic responses. When compared with other sensors, central processing of vestibular information is profoundly convergent, with vestibular nuclei receiving inputs from an array of cortical, cerebellar, and brainstem structures (reviewed in Cullen and Roy, 2004). In fact, the EVN receives polysynaptic innervation by autonomic centers including the hypothalamus, reticular formation, solitary nucleus, and raphe nucleus (Metts et al., 2006). Although, work is yet to explicitly consider the role of the EVN in sympathetic responses, its participation or influence remains plausible, particularly given vestibular involvement in the vestibulo-sympathetic pathway (Holstein et al., 2014, 2016). Indeed, vestibular activation has been shown to alter sympathetic efferent discharge arising from the thoracic spinal cord (Ray et al., 1997; Kerman et al., 2000; Zakir et al., 2000; Voustianiouk et al., 2006). As well, there is convincing evidence for vestibular participation in compensating for posture-related blood pressure changes in cats and humans (Yates and Miller, 1994; Kaufmann et al., 2002; Voustianiouk et al., 2006), which is likely via vestibular output to the diencephalon (Matsuda et al., 2004). In mice where auditory and vestibular hair cells failed to differentiate, normal physiological responses to hypergravtiy were absent (Murakami et al., 2002). This may implicate a role for vestibular hair cells as primary regulators of autonomic responses to elevated gravity levels. Given hair cell and afferent responses to EVS activation (see Section Vestibular Afferent and Hair Cell Responses to EVS Activation), and the extensive innervation of these end organs by the EVN neurons, it remains plausible that the EVS is not only activated but also functions in maintaining and regulating these reflexes.

Indeed, recent work has implicated central vestibular nuclei in the vestibulo-sympathetic pathway and reflex. Particularly, direct connections were shown between caudal vestibular nuclei, and the caudal and rostral ventrolateral medulla (Holstein et al., 2011), which partakes in vestibular-related blood pressure changes (Yates and Bronstein, 2005). Spinal, medial, and superior vestibular nuclei were also activated in response to sinusoidally-modulated galvanic vestibular stimulation that modulated blood pressure (Holstein et al., 2012). More recent work identified central vestibular nuclei participation in sympathetic blood pressure changes, but interestingly found no participation of the efferent vestibular pathway during the vestibuolosympathetic reflex (Holstein et al., 2014, 2016). Given that polysynaptic connections to the EVN were identified with autonomic centers such as the hypothalamus (Metts et al., 2006), it remains plausible that the EVS contributes to other sympathetic activities.

### Involvement of the EVS in top-down modulation of vestibular sensitivity

Most hypotheses regarding EVS function are concerned with the modulation of afferent discharge on a short time scale—that is, to modulate incoming afferent discharge for quick vestibular accommodation. Early reports of excited efferents and afferents during states of arousal in the oyster toadfish support this suggestion (Highstein and Baker, 1985; Boyle and Highstein, 1990). To implicate vestibular efferents in afferent discharge, Plotnik et al. (2002) obtained efferent-mediated responses by adjusting the head position of decerebrate and anesthetized chinchillas such that the innervated semicircular canal was placed at near right angles to the plane of motion, so that conventional rotational responses of vestibular afferent fibers were nulled (Plotnik et al., 2002). Unlike the variety of responses previously observed in semicircular canal afferents of decerebrate pigeons to contralateral canal stimulation (Dickman and Correia, 1993), the responses here were solely excitatory, and also considerably larger in irregular firing afferents in decerebrate when compared with anesthetized chinchilla (Plotnik et al., 2002). These type III responses (i.e., bidirectional excitatory rotational responses) resembled those obtained from electrical stimulation of efferent pathways and were abolished following vestibular nerve sectioning (Plotnik et al., 2002). In addition to these efferent-mediated rotational responses, Plotnik et al. (2005) also observed periodic fluctuations (up to 300 spikes/s) in the background discharge of irregular afferent fibers. The amplitude of fluctuations positively correlated to the size of individual afferent type III efferent-mediated rotational responses (Plotnik et al., 2002, 2005), hinting at an excitatory feed-forward positive feedback loop (Plotnik et al., 2005). Importantly, these fluctuations are not observed in alert-behaving animals (Louie and Kimm, 1976; Sadeghi et al., 2007, 2009). Nonetheless, efferent-mediated afferent rotational responses were indeed recorded in alert monkeys, but were typically small in irregular fibers, suggesting that vestibular efferents weakly act upon afferent discharge in the absence of high frequency shock trains (Sadeghi et al., 2009). Given the mutual excitation of afferents and efferents, these findings support a positive feedback loop. It is possible that this loop is mediated and modulated by higher centers and/or other systems to generate motor and vestibular coordination (for review, see Holt et al., 2011).

The EVS in the CNS could also serve in an auto-regulatory role with peripheral vestibular receptors, further giving merit to the suggestion of a feedback loop. A system of vestibular sensory auto regulation, which involves central and peripheral mechanisms, exists (Fitzpatrick and Watson, 2015). In healthy human subjects, vestibular perceptual and balance responses [assessed via galvanic vestibular simulation (GVS)] were measured before and after 10 min of imposed canal conditioning (stochastic yaw rotation) to explore central auto regulation of vestibular afferent activity during ambient motion (Fitzpatrick and Watson, 2015). The conditioning attenuated both reflexive and perceptual vestibular responses, while the threshold for detecting the imposed stimulus more than doubled and remained elevated for 30 min. Given the anatomical outlay of the EVS, that is the pre- and post- synaptic connections made with primary vestibular afferents, as well as receiving dendritic inputs from other vestibular nuclei and other sensory centers (Metts et al., 2006), Fitzpatrick and Watson (2015) implicate vestibular efferents in this auto regulation. Although, this functional hypothesis is in line with a feedback loop with vestibular afferents, there remains no direct connection. As well, these changes could occur at any stage of vestibular circuitry, be it central or peripheral.

### The EVS signals corollary discharge between vestibular and other CNS structures

The vestibular system is continuously bombarded with altering stimulus amplitudes and frequencies that it decomposes for use in orientation, posture, and spatial navigation (Straka and Dieringer, 2004; Angelaki and Cullen, 2008). In addition, active movement generates sensory reafference that can interfere with original extrinsic exafference signal transduction (Cullen, 2004; Cullen et al., 2011). The vestibular system as a whole therefore requires an adaptable neural processing circuitry to ensure the optimization and accuracy of mechanosensory signal detection and interpretation during motion (Carriot et al., 2014). A model that adaptively adjusts for this sensory encoding is corollary discharge, or efference copy, of the motor command (von Holst and Mittelstaedt, 1950). They serve to inform associated brain regions of impending movements and generate the expected sensory outcomes for overall sensorimotor transformation (Crapse and Sommer, 2008; Sommer and Wurtz, 2008). In context of vestibular coordination, efference copy signals are in a perfect position to influence the continuous fluctuations of vestibular signaling, both peripherally and centrally, and comply with the multimodal nature of the vestibular system. The EVS may behave as a conduit for corollary discharge and communications between other CNS structures (e.g., the cerebellum and/or the spinal cord) and the vestibular system.

To date, only one study has linked corollary discharge signals from the central pattern generator (CPG) circuitry in the spinal cord to vestibular efferent neurons, albeit in amphibians. In larva *Xenopus* frogs, Chagnaud et al. (2015) found evidence for EVN neuronal transmission of frequency, duration and amplitude components of locomotor CPG output to vestibular afferents, to attenuate their stimulus encoding during self-motion, by using semi-isolated *in vitro* preparations (Chagnaud et al., 2015). They showed that central anterior and posterior vestibular nerve (AVN and PVN, respectively) fibers are phase-coupled with ipsilateral spinal ventral roots, and out-of-phase with contralateral spinal ventral root discharge. By comparing the activity between fibers in the central and peripheral aspects of the vestibular nerve during fictive swimming (the tadpole correlate for tail-based swimming), Chagnaud et al. (2015) demonstrate that EVN fibers, and not neighboring afferent fibers, are indeed active during, and are rhythmically coupled with locomotion. Combined Ca^2+^ imaging and electrophysiological recordings of efferent activity during spinal CPG activity, showed similar Ca^2+^ dynamics in all recorded efferent neurons suggesting that all neurons participate in conveying locomotor corollary discharge to the periphery. Stepwise removal of spinal cord segments found that corollary discharge information originates from rostral spinal segments, but alteration in firing patterns and exclusive ipsilateral coupling following hemisection at the level of the obex, excluded input from the reticular formation. Although, earlier work in mice showed dendritic inputs from the reticular formation to the EVN (Metts et al., 2006). Presumably this is the result of species differentiation (amphibians and mammals), the polysynaptic nature of the viral tracing, or could a reflection of ipsilateral connections.

Moreover, paired recordings of afferent fibers during fictive swimming and rotational stimuli revealed a relationship between efferent firing and afferent encoding. Interestingly, the authors observed an ~45% diminished peak-to-peak amplitude of discharge modulation during locomotor CPG activity than before locomotion in vestibular afferent fibers, suggesting a considerable attenuation of their gain during locomotion. This work suggests that locomotor corollary discharge is delivered via vestibular efferents to the periphery in order to attenuate the sensitivity of stimulus encoding during self-motion (Chagnaud et al., 2015). In this way, the EVS is able to modify peripheral signal transduction and encoding in real time, and partake in sensory up-down channeling for multisensory postural coordination. Combined, this work clearly demonstrates a role of the EVS in corollary discharge during patterned locomotion, at least in amphibians *in vitro*. While it is possible that similar processes occur in mammals, this is yet to be demonstrated. Indeed, as the authors point out, the origin of the rhythmic locomotor and voluntary head movement corollary discharge signals are very different between larval *Xenopus* and monkeys, respectively, suggesting that both the nature and origin of motor programming can exert differential influence on sensory signaling (Chagnaud et al., 2015).

### The role of the EVS in vestibular plasticity

The EVS has also been implicated in vestibular plasticity, particularly regarding the vestibuloocular reflex (VOR). EVS signaling mediated by α9 nAChRs expressed at efferent vestibular synapses on hair cells, can elicit inhibitory responses in afferents (Elgoyhen et al., 1994; Hiel et al., 1996; Anderson et al., 1997; Holt et al., 2001; Zhou et al., 2013) (extensively reviewed in Jordan et al., 2013), while α6β2 nAChRs have been implicated in efferent-mediated afferent excitation of calyx/dimorphic neurons (Holt et al., 2015). It has been recently shown that α9 nAChRs may influence vestibular compensation following unilateral labyrinthectomy (Eron et al., 2015; Hübner et al., 2017). Given that the α9 subunit is expressed at EVN synapses, a missense mutation in the gene coding for this receptor subunit could compromise EVN output to the periphery. Indeed, the efficacy of the VOR was compromised in α9 nAChR knockout mice with ~70% reduction in vestibular adaptive ability (Hübner et al., 2015). Moreover, when compared to the baseline functional recovery of control mice following UL (~75% ipsilesional and ~90% contralesional), α9 nAChR knockout mice only regained ~30% ipsilesional and ~50% contralesional function (Hübner et al., 2017). These data implicate central and/or peripheral EVS mechanisms in VOR adaptability and compensation. However, there is also evidence that peripheral vestibular mechanisms (including vestibular afferent changes) do not play a role in vestibular compensation (Sadeghi et al., 2007), and that efferent activity does not play a role in VOR adaptability in awake behaving monkey (Miles and Braitman, 1980), with the latter suggesting that the adaptive mechanisms of the VOR reside within central circuits.

Along with ACh, CGRP is also co-expressed by EVN neurons and peripheral efferent terminals (Ohno et al., 1991; Luebke et al., 2014), and partakes in vestibular efferent and peripheral interaction. CGRP null mice demonstrated an ~50% decrease in VOR sensitivity (Luebke et al., 2014), further suggesting that disruption to normal EVN activity reduces the functionality and efficacy of the VOR. Interestingly, cholinergic staining of the same animals appeared normal (Luebke et al., 2014), with the authors suggesting chronic loss of CGRP from birth may contribute to compensatory mechanisms that mediate VOR plasticity. Of note however is the suggestion that CGRP in the EVN (as well as the auditory efferent system) instead plays a role in maturation of peripheral inner ear structures, for example by contributing to maturation of mechanical properties of the inner ear, or by tuning afferent responses (for review, see Simmons, 2002). Regardless, with this caveat in mind it remains reasonable to suggest that the function of the EVS could include some level of influence or participation in vestibular plasticity and compensation of the VOR. Further support for the EVS's role in the VOR comes from data regarding KCNQ potassium channels. The activation of mAChRs and closure of KCNQ potassium channels have been implicated in driving efferent-mediated slow afferent excitation in turtle (Holt et al., 2017). Interestingly, in KCNQ4 and KCNQ5 knockout mice, VOR performance again is impaired (Spitzmaul et al., 2013).

It should be noted that the EVN is not the only candidate to mediate compensation and habituation of the VOR, and has only been implicated in this function in recent literature (for example, Luebke et al., 2014; Hübner et al., 2015, 2017). Indeed, there is an extensive body of literature regarding the role of the nodulus and uvula in dynamic (i.e., gain) control of the VOR (some examples of which include Waespe et al., 1985; Torte et al., 1994).

## Summary

While incomplete, the functional role of most efferent systems in the nervous system has been characterized. In the vestibular system however, a conclusive functional role in mammals has yet to be confirmed. While there is a consensus regarding vestibular efferent morphology, location, and action on peripheral vestibular hair cells and primary afferents, a distinct functional role in motor and vestibular coordination had not yet been ascribed. Reasons for this include limited recordings from EVN neurons themselves, as well as a lack of understanding of EVS circuitry within the nervous system of any species. Recent work though has suggested potential functional roles. For example, the EVS has been implicated in efference copy generated from spinal cord circuitry during locomotion, while participation in vestibular plasticity and compensation has also been suggested. Older work has also demonstrated vestibular efferent activation following specific behavioral and environmental cues, suggesting a context dependent efferent activity. Given the unique nature of each of these different findings, it appears that the EVS functions distinctly under a diverse range of conditions. Investigations concerning direct monosynaptic inputs to the EVN would help establish the overall circuitry involved in EVS function by highlighting the direct connections the EVN makes with other parts of the brain and spinal cord. Such information could help establish the context/s within which the EVN is activated. Moreover, combining monosynaptic tracing technologies with electrophysiological recordings could further investigate the role of the EVS in corollary discharge signaling in mammals. For example, recent work showed that introducing rabies glycoprotein during *in vivo* patch recording in mouse layer 5 pyramidal cells, followed by glycoprotein-deficient rabies virus 2 days following resulted in the successful bridging of physiological and synaptic properties with anatomical and circuitry profiles of individually recorded neurons (Rancz et al., 2011). Alternatively, genetically encoded neural indicators of cellular activity can also be selectively expressed in neurons of interest (via viral techniques), and allow monitoring of their activity under different behavioral conditions *in vivo* (Perry et al., 2015). Targeted EVN manipulation (for example via optogenetic, electrophysiological or viral means), and subsequent behavioral testing, as well as direct recording or manipulation of EVN neurons and their inputs, could also expand hypotheses concerning EVS function.

## Author contributions

MM provided conceptual input, drafted the manuscript and created the figures. AC and AM provided conceptual and editorial input to the manuscript and figures.

### Conflict of interest statement

The authors declare that the research was conducted in the absence of any commercial or financial relationships that could be construed as a potential conflict of interest.
