# Corrigendum: Reviewing the Role of the Efferent Vestibular System in Motor and Vestibular Circuits

**DOI:** 10.3389/fphys.2018.00687

**Published:** 2018-05-30

**Authors:** Miranda A. Mathews, Aaron J. Camp, Andrew J. Murray

**Affiliations:** ^1^Sensory Systems and Integration Laboratory, Bosch Institute, Discipline of Biomedical Science, University of Sydney, Sydney, NSW, Australia; ^2^Sainsbury Wellcome Centre for Neural Circuits and Behaviour, University College London, London, United Kingdom

**Keywords:** efferent vestibular system, efferent vestibular nucleus, EVS, EVN, corollary discharge, VOR, vestibular, vestibular plasticity

In our original review article, there was a mistake in the reporting of Lysakowski and Singer (2000) in text and the placement of that publication in Figure 1. The original text on page 2 included the following statement:

**Figure 1:**
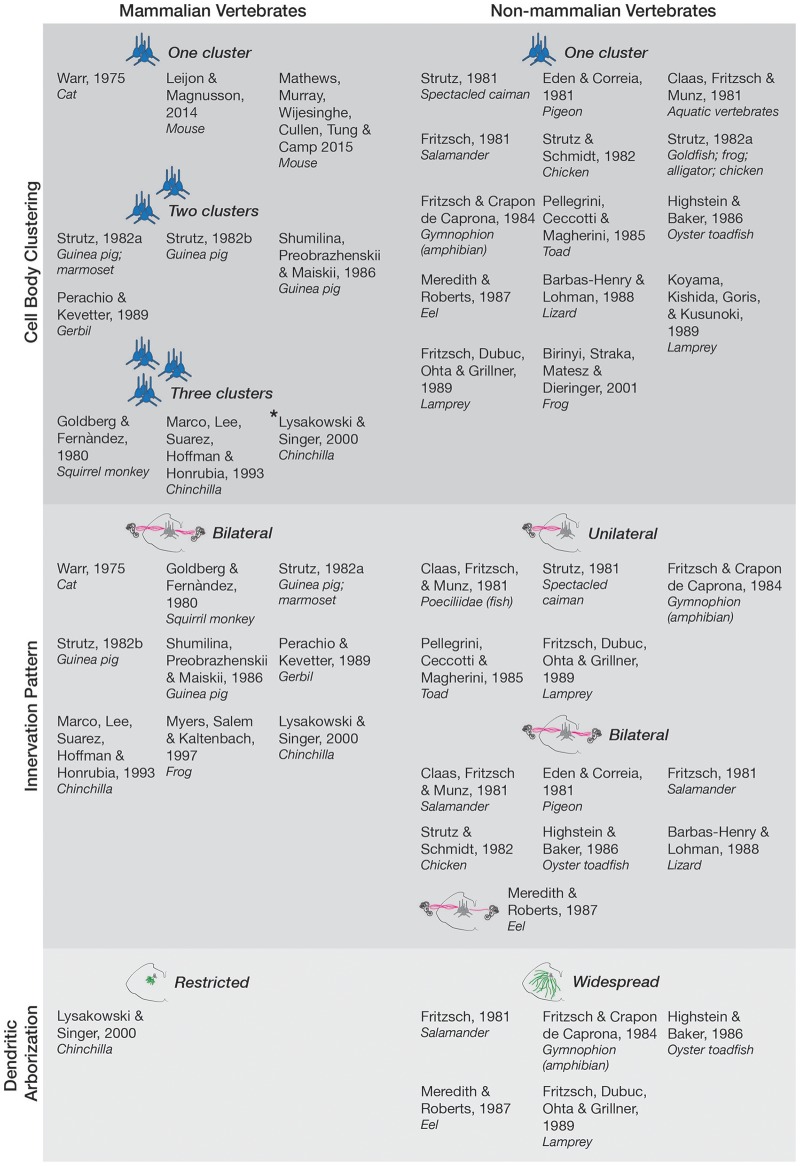
Anatomy and morphology of the EVS across vertebrates. Studies that directly investigated EVS anatomy and morphology were separated under the following categories-cell body clustering, innervation pattern, and dendritic arborization. Studies that assessed more than one category are mentioned in each respective category they investigated. Where more than one cell body cluster was observed, the number of clusters is labeled and depicted with the respective number of blue pictorial clusters. Asterisk next to Lysakowski and Singer (2000) denotes one cluster likely projecting to middle ear instead of peripheral vestibular labyrinth. Uni- and bilateral projections are also labeled and depicted with pink lines from a coronal brainstem schematic out towards the inner ear (drawings not to scale). Only one bilateral projection is drawn for Meredith and Roberts (1987) *eel* as they denoted it as a minor finding. Expansive green lines along the brainstem tegmentum denote widespread arborization of dendrites, and shorter green lines depict restricted arborization, as labeled. Nonmammalian species included all animals groups not classified as mammals.

“Studies in chinchilla present a variable picture from a single EVN cluster (Lysakowski and Singer, 2000) to three anatomically distinct groups near the facial nerve, abducens nerve, and vestibular nuclei (Marco et al., 1993).”

has been modified to:

“In other mammalian studies, more than one cluster was observed with the major nucleus being referred to as group *e* (Goldberg and Fernàndez, 1980), located dorsal and/or ventral to the facial nerve (Shumilina et al., 1986; Perachio and Kevetter, 1989). Smaller clusters are scattered in the caudal pontine reticular nucleus and the medial reticular nucleus (Strutz, 1982a,b). Interestingly, in the chinchilla, three anatomically distinct groups near the facial nerve, abducens nerve, and vestibular nuclei were distinguished (Marco et al., 1993; Lysakowski and Singer, 2000), though the cluster ventral to the facial nerve likely reflects projections to the middle ear rather than the peripheral vestibular labyrinth (Lysakowski and Singer, 2000).”

Figure 1 has also been amended in line with this modification, as well as a typographical correction of “*squirril monkey”* to “*squirrel monkey”* under (Goldberg and Fernàndez, 1980) in the “Three Clusters” section. The figure legend has also been amended to clarify these changes. The amended Figure 1 is now:

Figure 1 legend has been modified from:

“Figure 1. Anatomy and morphology of the EVS across vertebrates. Studies that directly investigated EVS anatomy and morphology were separated under the following categories—cell body clustering, innervation pattern, and dendritic arborization. Studies that assessed more than one category are mentioned in each respective category they investigated. Where more than one cell body cluster was observed, the number of clusters is labeled and depicted with the respective number of blue pictorial clusters. Uni- and bi-lateral projections are also labeled and depicted with pink lines from a coronal brainstem schematic out toward the inner ear (drawings not to scale). Only one bilateral projection is drawn for Meredith and Roberts (1987) eel as they denoted it as a minor finding. Expansive green lines along the brainstem tegmentum denote

widespread arborization of dendrites, and shorter green lines depict restricted arborization, as labeled. Non-mammalian species included all animals groups not classified as mammals.”

to:

“Figure 1. Anatomy and morphology of the EVS across vertebrates. Studies that directly investigated EVS anatomy and morphology were separated under the following categories—cell body clustering, innervation pattern, and dendritic arborization. Studies that assessed more than one category are mentioned in each respective category they investigated. Where more than one cell body cluster was observed, the number of clusters is labeled and depicted with the respective number of blue pictorial clusters. Asterisk next to Lysakowski and Singer (2000) denotes one cluster likely projecting to middle ear instead of peripheral vestibular labyrinth. Uni- and bi-lateral projections are also labeled and depicted with pink lines from a coronal brainstem schematic out towards the inner ear (drawings not to scale). Only one bilateral projection is drawn for Meredith and Roberts (1987) eel as they denoted it as a minor finding. Expansive green lines along the brainstem tegmentum denote widespread arborization of dendrites, and shorter green lines depict restricted arborization, as labeled. Non-mammalian species included all animals groups not classified as mammals.”

The authors sincerely apologize for the ambiguities. These changes do not significantly alter the review article.

The original article has been updated.

## Conflict of interest statement

The authors declare that the research was conducted in the absence of any commercial or financial relationships that could be construed as a potential conflict of interest.
